# Secretory phospholipase A2 in the pathogenesis of acute dengue infection

**DOI:** 10.1002/iid3.135

**Published:** 2016-12-11

**Authors:** Chandima Jeewandara, Laksiri Gomes, Sukhitha Udari, S.A. Paranavitane, N.L.A. Shyamali, Graham S. Ogg, Gathsaurie Neelika Malavige

**Affiliations:** ^1^ Centre for Dengue ResearchUniversity of Sri JayawardanapuraSri Lanka; ^2^ Faculty of Medical SciencesDepartment of MedicineUniversity of Sri JayawardanapuraSri Lanka; ^3^ MRC Human Immunology UnitNIHR Biomedical Research CentreWeatherall Institute of Molecular MedicineOxford

**Keywords:** mast cell tryptase, platelet activating factor, secretory phospholipase, severe dengue, vascular leak

## Abstract

**Introduction:**

Platelet activating factor (PAF) is an important mediator of vascular leak in acute dengue. Phospholipase A2s (PLA2) are inflammatory lipid enzymes that generate and regulate PAF and other mediators associated with mast cells. We sought to investigate if mast cell activation and increases in secretory sPLA2s are associated with an increase in PAF and occurrence of dengue haemorrhagic fever (DHF).

**Methods:**

The changes in the levels of mast cell tryptase, PAF and the activity of sPLA2 were determined throughout the course of illness in 13 adult patients with DHF, and 30 patients with dengue fever (DF).

**Results:**

We found that sPLA2 activity was significantly higher in patients with DHF when compared to those with DF, during the first 120 h of clinical illness. sPLA2 activity was significantly associated with PAF levels, which were also significantly higher in patients with DHF. Although levels of mast cell tryptase were higher in patients with DHF, the difference was not significant, and the levels were not above the reference ranges. sPLA2 activity significantly correlated with the degree of viraemia in patients with DHF but not in those with DF.

**Conclusion:**

sPLA2 appears to play an important role in the pathogenesis of dengue. Since its activity is significantly increased during the early phase of infection in patients with DHF, this suggests that understanding the underlying mechanisms may provide opportunities for early intervention.

## Introduction

Dengue is one of the most important mosquito borne virus infections in the world, affecting approximately 390 million individuals annually 1. It has been estimated that in 2013, 58.4 million individuals developed symptomatic dengue virus infection, of whom 18% required hospitalization 2. Although mortality rates have improved due to better management, dengue is associated with significant morbidity with an annual cost estimated to be US$ 8.9 billion 2.

Typical dengue is characterised by fever, myalgia, arthralgia and gastrointestinal symptoms such as abdominal pain and vomiting 3. In some patients, this initial febrile or viraemic phase is followed by a critical phase, which is associated with increased vascular permeability 4. This is evident by a rise in the haematocrit, reduced pulse pressure, pleural effusions, ascites and shock 5. The critical phase typically lasts for 24–48 h after which most patient proceed to recovery.

Many inflammatory mediators have been implicated in the vascular leak seen in acute dengue 6, 7, 8. In an earlier study, we found that levels of platelet activating factor (PAF) were significantly higher in patients with dengue haemorrhagic fever (DHF) when compared to those with dengue fever (DF), especially during the critical phase 9. In endothelial cells, the sera obtained from patients with acute dengue caused a reduction of the trans‐endothelial resistance and tight junction protein expression, both of which were significantly inhibited by PAF receptor blocker 9. Since these experiments confirmed that PAF was likely to be an important mediator of vascular leak, we sought to identify mechanisms known to regulate PAF, in order to find new therapeutic targets, in the treatment of acute dengue.

PAF is an inflammatory lipid mediator that is produced by mast cells, monocytes, macrophages, neutrophils, endothelial cells and platelets 10, 11. Phospholipase A2 and acetyltransferase are required for its synthesis 12. Phospholipase A2s constitute a group of inflammatory lipid enzymes that act on cellular phospholipids to generate free fatty acids (e.g. PAF) and lysophospholipids and are known to enhance the systemic inflammatory response 12, 13. Both secretory phospholipase A2s (sPLA_2_) and cytoplasmic phospholipase A2 are known to generate and regulate PAF and both are produced by mast cells, endothelial cells, hepatocytes, monocytes and many types of epithelial cells 14 and are degraded following binding with specific binding proteins 12, 13, 15. Endothelial cells are also known to produce PAF and it has been shown that mast cell products such as vascular endothelial growth factor (VEGF) induces production of PAF through the action of sPLA2s 16. Since VEGF has been found to be elevated especially in patients with DHF and to associate with vascular leak 17, 18, VEGF could be inducing PAF through activation of sPLA2s. The activity of both cPLA2 and sPLA2 are also induced by inflammatory cytokines such as TNFα, IL‐1β and IL‐6 13, 19. Since these cytokines are known to be highly elevated, especially in patients with DHF and follow the same patterns of production as PAF 20, they could also be inducing the activity of the lipid enzymes and subsequent PAF production.

Mast cells are activated occurs in acute dengue and mast cell mediators have been associated with vascular leak in mouse models of this disease 8, 18, 21. Mast cell products such as VEGF, mast cell tryptase and chymase levels have been shown to be significantly higher in patients with more severe forms of dengue and VEGF could be acting through induction of sPLA2s to simulate production of PAF 17. In vitro studies have shown that mast cells are directly infected with the dengue virus (DENV) 22. Antibody dependant enhancement (ADE) has shown to significantly increase the release factors that lead to endothelial activation in in vitro studies 23 and in dengue mouse models 8. DENV infection of mast cells is potentiated by ADE and is facilitated by autophagy 24. In addition, pre‐existing DENV specific antibodies contribute to disease severity by inducing mast cell degranulation through Fcγ receptors 8. Many studies have shown that mast cell mediator production is increased in patients with more severe forms of disease and DENV‐specific antibodies contribute to disease pathogenesis by ADE and by inducing mast cell degranulation. By contrast, some studies have shown that mast cells might be protective in acute dengue. For instance, a recent study showed that DENV infection in mice deficient in mast cells had higher viral loads, prolonged viraemia and increased bleeding 25. These disparate findings could be reconciled if mast cell activation is important for controlling DENV replication but also contributes to immune mediated pathology.

Since mast cells are an important source of PAF and mast cell activation and degranulation have been shown to contribute to vascular leak and disease severity, we investigated the relationship between PAF and mast cell tryptase during the course of illness in patients with DHF and DF and also in those with primary and secondary dengue. In addition, since PLA2 is known to regulate PAF and since mast cells are also a known source of certain subgroups of this lipid enzyme, we also evaluated the kinetics of sPLA_2_ activity in these patients.

We found that during the first five days (120 h) of clinical illness, serum sPLA_2_ activity was significantly higher in patients with DHF, when compared to those with DF. Although, mast cell tryptase levels were higher in patients with DHF, this increase was not statistically significant. In addition, both the activity of sPLA_2_ and mast cell tryptase levels were significantly associated with the degree of viraemia in patients with DHF. Therefore, events that occur in early in DENV infection are likely to contribute to subsequent disease severity, and this suggests that these could be a focus of therapeutic targeting.

## Methods

We enrolled 43 adult patients with clinical features of dengue, admitted to a general medical ward in a tertiary care hospital (Colombo South Teaching Hospital) in Colombo during the year 2015. Following informed written consent, serial blood samples were obtained in the morning (5.00 a.m.) and afternoon (3.00 p.m.), from the time of admission to the time of discharge from hospital, throughout the course of their illness. Only those whose duration from onset of illness was ≤ 5 days were recruited. The day in which the patient first developed fever was considered as day one of illness. Those who were known to have chronic liver disease, chronic renal disease, pregnant individuals, those on a steroid dose of > 40 mg/day for over seven days were excluded from the study.

Clinical features including fever, blood pressure, pulse pressure and the urine output were measured at least four times a day. The severity of acute dengue was classified according to the 2011 WHO dengue guidelines 3. Accordingly, patients who had a rise in haematocrit above 20% of the baseline haematocrit or detectable fluid in the pleural or abdominal cavities by ultrasound scanning, were classified as having DHF. Shock was defined as having cold clammy skin, along with a narrowing of pulse pressure of ≤ 20 mmHg. Based on the 2011 WHO guidelines, 30 patients had DF and 13 had DHF. As patients with DHF were in hospital for a longer time than those with DF, approximately five to nine serial blood samples were collected from this group, compared to four to five samples from those with DF.

### Ethics statement

The ethical approval was granted from the Ethical Review Committee of the University of Sri Jayawardanapura.

### Quantitative PAF and mast cell tryptase assays

Levels of PAF and mast cell tryptase were determined in all serial blood samples which were obtained twice a day. All the assays were done in duplicate. The PAF (Cusabio, Wuhan, China) and mast cell tryptase assays (Abbexa, Cambridge, UK) were carried out using a quantitative ELISA according to manufacturers’ instructions. These assays have been used previously to quantify levels of serum PAF 9 and mast cell tryptase.

### Assays for sPLA_2_ activity in serum

The activity of sPLA_2_in patients with dengue infections were measured using a commercial sPLA2 kit (Abcam, Cambridge, UK), according to manufacturer's instructions. Briefly in a flat‐bottom 96‐well plate, 10 μl DTNB and 15 μl of the assay buffer were added to the blank wells and 10 μl DTNB, 10 μl Bee Venom PLA2, and 5 μl assay buffer added to the positive control wells. Ten micro litres DTNB, and 5 μl assay buffer along with 10 μl of the serum sample were added to each sample well. The reaction was initiated by adding 200 μl Substrate Solution to each well. All the samples were tested in duplicate. The absorbance was read every min at 414 (or 405) nm using an ELISA plate reader to obtain at least five time points. The reaction rate was determined using a DTNB extinction coefficient of 10.66 mM‐1 according to the recommended formula for calculating the sPLA2 activity.

### Determining viral loads in serial blood samples

RNA was extracted from all serial serum samples using QIAamp Viral RNA Mini Kit (Qiagen, Valencia, CA) according to the manufacturer's protocol. The RNA was reverse transcribed into cDNA in GeneAmp PCR system 9,700 using High Capacity cDNA reverse transcription kit (Applied Biosystems, Foster City, CA) according to the manufacturer's instructions. Reaction conditions were 10 min at 25°C, 120 min at 37°C, 5 min at 85°C and final hold at 4°C.

Multiplex quantitative real‐time PCR was performed as previously described using the CDC real time PCR assay for detection of the DENV 26. This assay was modified to quantify the DENV apart from quantitative analysis. Oligonucleotide primers and a dual labelled probe for DEN 1,2,3 and 4 serotypes were used (Life technologies, Bengaluru, India) based on published sequences 26. The probe was dual‐labelled with the probe and the QSY quencher. The DENV serotype specific primers were labelled as follows: DENV‐1 with JUN, DENV‐2 with ABY, DENV‐3 with FAM, DENV‐4 with VIC. The reactions consisted of 20 µl volumes containing the following reagents, TaqMan multiplex master mix (containing mustag dye), 900 nM of each primer, 250 nM of each probe, 2 µl of cDNA and nuclease free water (Applied Biosystems, USA). The reaction was performed in an Applied Biosystems7500, 96‐well plate detection system. Following initial denaturation for 20 s at 95°C, the reaction was carried out for 40 cycles of 3 s each at 95°C and 30 s at 60°C.The threshold cycle value (Ct) for each reaction was determined by manually setting the threshold limit. Virus quantification (pfu/mL) of unknown samples was performed using the standard curve.

### Generating standard curves for quantifying the DENV

To generate standard curves, the four DENVs were grown in C6/36 cell lines supplemented with L15 media at 28°C. Virus supernatants were harvested seven following infection and immediately used to infect vero81 cell lines. To determine the infective virus particles by plaque assays, virus culture supernatants were serially diluted and inoculated in triplicate. Undiluted sample from the virus culture supernatant was used as the positive control and culture media as the negative control. After 5 days of incubation at 37°C in 5% CO_2_ incubator, the plaques were developed and counted. Virus concentrations were calculated as pfu/mL. Following quantification of the viruses, the standard curves were generated as serial dilutions from 10^6^ to 10^1^ of pfu/mL for each virus serotype. In order to quantify the virus in clinical samples the unknowns were compared to known values in the standard curves of each virus. The viral loads were expressed as pfu/mL.

### Detection of dengue NS1 antigen and dengue specific antibodies

Acute dengue infection was confirmed in the serum samples using the NS1 early dengue ELISA (Panbio, Brisbane, Australia). All assays were done in duplicate. Dengue antibody response was also confirmed in these patients with a commercial capture‐IgM and IgG enzyme‐linked immunosorbent assay (ELISA) (Panbio). The ELISA was performed and the results were interpreted according to the manufacturers’ instructions. This ELISA assay has been validated as both sensitive and specific for primary and secondary dengue virus infections 27, 28.

### Statistical analysis

Statistical analysis was performed using Graph PRISM version 6. As the data were not normally distributed (as determined by the frequency distribution analysis of Graphpad PRISM), non‐parametric tests were used in the statistical analysis. Differences in the serial values of sPLA2, mast cell tryptase, PAF and viral loads in patients with DHF and DF were done using multiple unpaired t tests. Corrections for multiple comparisons were done using Holm‐Sidak method and the statistical significant value was set at 0.05 (alpha). The association between the PAF, sPLA2, mast cell tryptase and viral loads was done using Spearman correlation.

## Results

Of the 43 patients with acute dengue, 30 were classified as having DF and 13 were classified as having DHF based on the WHO 2011 dengue guidelines 3. None of the patients developed shock or severe bleeding manifestations and all of them eventually recovered. A total of 36/43 (80.7%) of patients were infected with DENV‐1 while 1/43 (2.3%) were infected with DENV‐4. Six of the patients were negative by quantitative PCR although their NS1 test was positive and dengue specific IgM and IgG were positive. The average duration of illness when obtaining the first blood sample was 93.7 (SD ± 18.9) h since the onset of illness.

### Kinetics of sPLA2 in the course of acute dengue infection

As in our previous studies we found that PAF was significantly increased in patients with DHF when compared to those with DF and the PAF levels were highest during the critical phase of dengue 9, we proceeded to investigate the kinetics of sPLA2 and mast cell tryptase compared to the kinetics of PAF in acute dengue infection.

**Figure 1:**
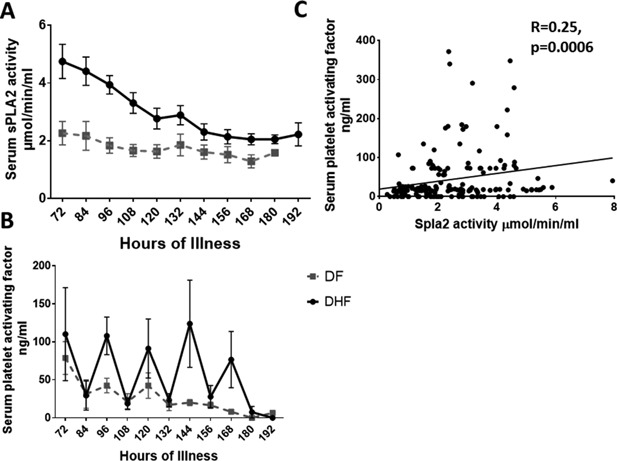
Kinetics of sPLA2 and PAF in patients with acute dengue infection. A: Serum sPLA2 activity was measured in serial blood samples in 13 patients with DHF and 30 patients with DF using a colorimetric assay. The mean and the standard error of mean (SEM) are shown. B: Levels of serum PAF were measured in serial blood samples in 13 patients with DHF and 30 patients with DF. The mean and the standard error of mean (SEM) are shown. C: Relationship between serum sPLA2 activity and levels of PAF levels in patients with DF and DHF.

Phospholipase A2s are a group of lipid enzymes, which act on cellular phospholipids to generate free fatty acids (e.g. PAF) and lysophospholipids 12. Since PAF was found to be a cause of vascular leak in acute dengue, we determined the kinetics of sPLA2, to determine if the changes in sPLA2 activity were similar to the changes in PAF. We found that sPLA2 activity was significantly increased in patients with DHF during the early phases of illness and then tends to reduce around 132–144 h of illness (Fig. 1A). sPLA2 activity was significantly higher in those with DHF when compared to those with DF during the first 120 h of illness. Although sPLA2 levels were still higher in patients with DHF when compared to those with DF beyond 120 h of illness, this was not significant.

The kinetics of PAF were similar to our previous observations and the levels of PAF were significantly higher at 96 (*P* = 0.005) and 144 h of illness (*P* = 0.01) in patients with DHF when compared to those with DF (Fig. 1B). Similar to what we observed previously 9, the PAF levels showed a biphasic pattern with the levels been highest in the mornings and decreased to almost 0 undetectable by the afternoon. The levels of PAF gradually declined in patients with DF after 120 h of illness, whereas high levels of PAF were seen up to 168 h of illness in patients with DHF. sPLA2 activity significantly correlated with PAF levels in patients with acute dengue infection (Spearmans’ *r* = 0.25, *P* = 0.0006) (Fig. 1C).

Activity of sPLA2 also significantly correlated with viral loads (Spearmans’ *r* = 0.19, *P* = 0.02) and inversely correlated with lymphocyte numbers (Spearmans’ *r* = −0.15, *P* = 0.04). On further analysis, the association of viral loads with serum sPLA2 activity was significant in patients with DHF (Spearmans *r* = 0.43, *P* = 0.001), but not in those with DF (Spearmans *r* = −0.22, *P* = 0.14). We did not observe any relationship between the viral loads with PAF in either patients with DF or with DHF. Serum sPLA2 activity also associated with liver transaminases as it significantly correlated with levels of serum aspartate transaminase levels (Spearmans *r* = 0.23, *P* = 0.005) and with serum alanine transaminase levels (Spearmans *r* = 0.23, *P* = 0.03).

### Kinetics of mast cell tryptase in acute dengue

**Figure 2:**
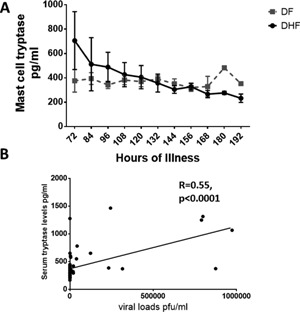
Kinetics of mast cell tryptase in patients with acute dengue infection Kinetics. A: Serum mast cell tryptase was measured in serial blood samples in 13 patients with DHF and 30 patients with DF. The mean and the SEM are shown. B: Relationship between serum mast cell tryptase levels and the degree of viraemia in patients with DHF (*n* = 13).

Mast cells have recently been shown to be important in the pathogenesis of dengue and certain mast cell mediators have been associated with vascular leak 22, 29. Amongst other cells, mast cells are an important source PAF and some groups of sPLA2s have been shown to be important in maturation of mast cells 30, 31. Since we found that both sPLA2 activity and PAF levels were higher in DHF, we investigated if this increase was associated with activation of mast cells. As tryptase is highly specific for mast cells 11, we proceeded to determine if mast cell degranulation, as indicated by release of tryptase is associated with high levels of PAF and increased activity of sPLA2. During the 108 h of illness, mast cell tryptase levels were found to be increased in patients with DHF when compared to those with DF, although this was not significant at any time point (Fig. 2A). The normal ranges for mast cell tryptase in healthy individuals is considered to be < 11.5 ng/mL (11,500 pg/mL) 32. None of the patients with DHF had mast cell tryptase levels higher than 11.5 ng/mL at any time point during illness. In addition, mast cell tryptase did not correlate with activity of sPLA2 (Spearman’ r = 0.06, *P* = 0.4) or with levels of PAF (Spearmans *r* = −0.01, *P* = 0.87). However, mast cell tryptase was associated with the degree of viremia (Spearman's *r* = 0.31, *P* = 0.0002). This association of mast cell tryptase with the degree of viraemia was significant in those with DHF (Spearmans *r* = 0.55, *P*< 0.0001) (Fig. 2B), whereas no such association was seen in those with DF (Spearmans *r* = 0.05, *P* = 0.62). Mast cell tryptase also did not associate with any of the laboratory disease severity markers such as liver enzymes, lymphocyte or platelet counts.

### PAF, sPLA2 and mast cell tryptase in those with primary and secondary dengue infection

**Figure 3:**
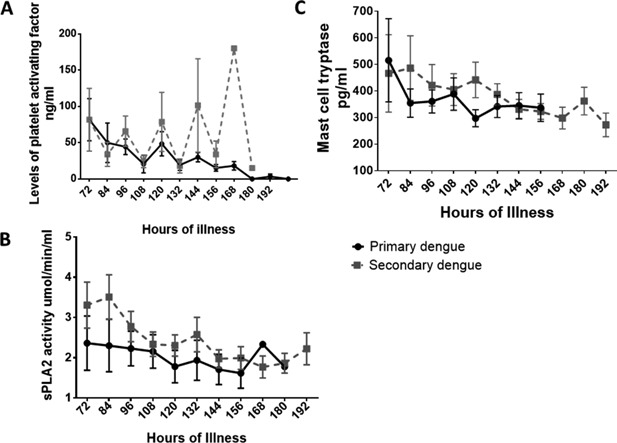
Kinetics of PAF, sPLA2 and mast cell tryptase in patients with primary and secondary.A: PAF levels were measured in serial blood samples in 29 patients with secondary dengue and 14 patients with primary dengue. The mean and the SEM are shown. B: Serum sPLA2 activity was measured in serial blood samples in 29 patients with secondary dengue and 14 patients with primary dengue. The mean and the SEM are shown. C: Mast cell tryptase levels were measured in serial blood samples in 29 patients with secondary dengue and 14 patients with primary dengue. The mean and the SEM are shown.

DHF is more common is secondary dengue infection, and this is thought to be due to enhancement of infection in Fc receptor bearing cells, caused by DENV‐specific poorly‐neutralising, highly cross‐reactive antibodies 33, 34. Pre‐existing DENV‐specific antibodies have also been shown to increase mast cell degranulation in both in vitro and in dengue mouse models 8. Therefore, we determined the changes in the kinetics of mast cell tryptase, activity of sPLA2 and levels of PAF in patients with primary and secondary dengue. Of the 14 patients had primary dengue and 29 patients had a secondary dengue. Of the 13 patients with DHF only four had a primary dengue. Therefore, of the 14 patients with primary dengue, four developed DHF and 10 developed DF. Of the 29 patients with secondary dengue, nine developed DHF and 20 developed DF. We did not observe significant differences in the kinetics of mast cell tryptase, sPLA2 or PAF in those with primary or secondary dengue (Fig. 3).

## Discussion

In this study we investigated the changes in the activity of sPLA2 and levels of PAF and mast cell tryptase throughout the course of illness in patients with varying severity of dengue infection. We found that serum sPLA2 activity was markedly increased and significantly higher in patients with DHF, during the first 120 h from the onset of illness. Levels of PAF were also significantly higher in patients with DHF at 96 and 144 since the onset of illness. Although, the mast cell tryptase levels were also higher in patients with DHF, this was not significant at any time point since the onset of illness.

Phospholipase A2s are a group of lipid enzymes, which act on cellular phospholipids to generate free fatty acids and lysophospholipids and also generate PAF 12. Over one third of the phospholipases are of the secretory form (sPLA2), which has 10 isoforms 14. Among the main isoforms sPLA2, group IIA is the main isoform detected in serum and its activity has been shown to be induced by many proinflammatory stimuli including bacterial products 14, 35. Due to its high affinity for bacterial membrane lipids, it effectively degrades many bacteria. However, many inflammatory lipid mediators and arachidonic acid metabolites are also generated during the activity of sPLA2 and therefore, the group IIA sPLA2s are also known as inflammatory phospholipase enzymes with wide ranging effects 13, 14, 36, 37. We found that in patients with acute dengue, the sPLA2 activity was highest very early in the course of infection and the levels were significantly higher in those with DHF. The activity of sPLA2 diminished towards 120–132 h of illness and from this time onwards, there was no difference between the activity of sPLA2 in patients with DHF and those with DF. Interestingly, the degree of viraemia significantly correlated with the sPLA2 activity in those with DHF but not in those with DF. Since in acute dengue infection the viraemia also tends to decline with time and become undetectable and very low at around 120–132 h, it is possible that the virus itself or certain viral proteins such as NS1 could be inducing the activity of sPLA2. Recently it was shown that dengue NS1 has a LPS like activity and acts through TLR4 receptors to induce production of inflammatory cytokines from monocytes and macrophages 38, 39. NS1 was also shown to induce vascular leak in both in vitro and in dengue mouse models 38, 40. The mechanisms by which dengue NS1 induces vascular leak are not well studied 40 but may involve complement mediated effects. However, NS1 possibly could induce sPLA2, thereby regulating PAF, which in turn induces vascular leak. Alternatively PAF may influence production of sPLA2 35. In our previous studies 9, significantly higher PAF levels were detected in patients with DHF and the dengue NS1 antigen was also shown to persist for a longer duration at higher levels 39, 41. Therefore, whether NS1 plays a role in inducing sPLA2 or PAF should be further investigated.

Activation of mast cells directly by the DENV and by DENV‐specific antibodies has been shown to occur in both in vitro and in dengue mouse models 8, 21, 23, 24. Mast cell products such as VEGF and chymase have been shown to be significantly elevated in patients with more severe forms of dengue infection 17, 21. Leukotriene receptor antagonists were found to significantly reduce the vascular leak in dengue mouse models 21. Since the accumulating evidence of the importance of mast cells in the pathogenesis of acute dengue infection, we investigated the changes of another mast cell specific protease, mast cell tryptase in patients with acute dengue. Although, the levels of mast cell tryptase were higher in patients with DHF, the levels were not significantly higher than patients with DF at any time point. In addition, although the mast cell tryptase levels were higher, they were not higher than the normal ranges observed in healthy individuals at any time point during acute dengue infection. As with the relationship with the activity of sPLA2 and the degree of viraemia, we found a significant correlation with the degree of viraemia and mast cell tryptase levels in patients with DHF, but not in those with DF. Although, St John et al. found that serum chymase levels inversely correlated with the viral loads in patients with DHF 21, this difference is possibly due to the fact that they studied the chymase levels and viral loads at one time point during illness, whereas our observations are of correlation between viral loads and tryptase throughout the course of illness.

DENV specific antibodies have shown to induce activation of mast cells by facilitating autophagy and also by inducing mast cell degranulation through the FcRγ receptors 8, 24. Therefore, activation and degranulation of mast cells due to pre‐existing DENV specific antibodies is thought to be another mechanism by which DENV‐specific antibodies contribute to severe clinical disease in secondary dengue infections 8. However, since none of the mast cell proteases such as tryptase or chymase themselves induce increased vascular permeability from endothelial cell lines, we determined the changes in the levels of mast cell tryptase, PAF and serum sPLA2 activity in patients with primary and secondary dengue. We did not find any difference in either levels of PAF or mast cell tryptase or in sPLA2 activity in those with primary and secondary dengue, during the course of illness. Although risk of DHF is known to be higher in those with secondary dengue infection 42, 43, 44, many individuals with primary dengue infection too have had DHF 45, 46, 47, which can be associated with fatalities. In fact some studies have shown that the likelihood of developing clinically inapparent dengue infection was similar during primary and secondary dengue infections 48. Therefore, the presence of pre‐existing DENV specific antibodies alone does not appear to solely explain severe secondary clinical disease, and other modifying factors are important. Since we found that PAF was an important mediator of vascular leak and since PLA2 activity induces production of PAF and other inflammatory mediators such as arachidonic acid metabolites including leukotrienes, it would be crucial to understand the pathways that lead to increased activity of sPLA2.

In summary, we found that the serum sPLA2 activity was significantly increased in patients with DHF and that sPLA2 activity was also associated with other disease severity markers such as liver transaminases. As previously observed, levels of PAF were significantly higher in patients with DHF and serum sPLA2 activity was found to correlate with PAF. However, since mast cell tryptase levels were not significantly higher in patients with DHF nor in those with secondary dengue, this suggests that non‐mast cell sources of PAF may also be important. Since sPLA2 activity was highest during early phase of clinical disease and since it significantly associated with the degree of viraemia, it would be important to understand the triggers that increase sPLA2 activity in order to develop new therapeutic targets.

### Conflicts of Interest

None declared.
